# Association between GDF-15 levels and changes in vascular and physical function in older patients with hypertension

**DOI:** 10.1007/s40520-016-0636-0

**Published:** 2016-10-12

**Authors:** Maryam Barma, Faisel Khan, Rosemary J. G. Price, Peter T. Donnan, C. Martina Messow, Ian Ford, Alex McConnachie, Allan D. Struthers, Marion E. T. McMurdo, Miles D. Witham

**Affiliations:** 1 Division of Cardiovascular and Diabetes Medicine, University of Dundee, Dundee, UK; 2 Dundee Epidemiology and Biostatistics Unit, University of Dundee, Dundee, UK; 3 Robertson Centre for Biostatistics, University of Glasgow, Glasgow, UK; 4 Ageing and Health, Ninewells Hospital, Dundee, UK

**Keywords:** Ageing, Biomarker, Cardiovascular disease, Frailty, Inflammation

## Abstract

**Background:**

Growth differentiation factor-15 (GDF-15) may be a biomarker of disease, protective response and/or prognosis, in older people with hypertension.

**Aims:**

To correlate baseline GDF-15 levels with physical and vascular health data in this population.

**Methods:**

Baseline blood samples were analysed using a GDF-15 ELISA assay kit. Correlations with baseline and 12-month outcome data, including measures of physical and vascular function, were performed.

**Results:**

A total of 147 individuals, mean age 76.8 ± 4.7 years, were included. 77 (52 %) were male. Baseline log_10_GDF-15 showed significant correlations with age (*r* = 0.37, *p* < 0.001), total cholesterol (*r* = −0.33, *p* < 0.001) and 6-min walking distance (*r* = −0.37, *p* < 0.001). Age remained significantly associated with log_10_GDF-15 in multivariable analysis (beta = −0.29, *p* = 0.001). Baseline log_10_GDF-15 was significantly associated with decline in 6-min walk distance over 12 months (beta = −0.27, *p* = 0.01) in multivariable models. No significant correlations were seen with changes in vascular function over 12 months.

**Conclusion:**

Baseline GDF-15 predicts declining physical, but not vascular, function in our population.

## Introduction

The decline in physical function that commonly accompanies ageing is multifactorial in aetiology. Impairments in multiple organ systems, including muscle, nerve, bone, blood vessels and the brain, all contribute to this loss in function. Cardiovascular disease (CVD) in particular is a common cause of hospital admissions amongst older patients and is also a key driver of functional decline. Functional loss, and the accompanying vulnerability to stressors, is captured in the concept of frailty [1]. It is clear that in order to effectively manage frailty, an approach that protects, or bolsters function, across multiple organ systems—i.e. an approach that builds resilience—is necessary [2]. For such an approach to be practical, interventions that target common pathologies affecting multiple organ systems are required; targets such as chronic inflammation, oxidative stress and mitochondrial dysfunction are examples of this. Both CVD and sarcopenia (the age-related decline in muscle function) have been linked to chronic inflammation. Effective interventions may be best deployed early in the process of decline, and to detect those at risk, markers that predict decline before the onset of frailty would be useful.

GDF-15 may represent a potential biomarker of age-related disease. Discovered in 1997, it is classified structurally as a divergent member of the transforming growth factor β (TGF-β) superfamily of cytokines [3]. This family comprises both pro- and anti-inflammatory molecules implicated in diverse inflammatory and fibrotic conditions. Activated macrophages produce GDF-15, and it is believed to serve a self-regulatory function by limiting macrophage activation. It is ubiquitously expressed, and levels rise with age, perhaps reflecting its role in regulating cell proliferation, apoptosis and repair, all of which constitute basic cellular stress responses [4, 5]. It may also affect energy metabolism and storage [6], showing correlations with BMI, inflammatory conditions (e.g. rheumatoid arthritis) and malignancies [7, 8]. A persistent, low-grade pro-inflammatory state is thought to be a key pathophysiological mechanism underlying ageing [9]. Given this, and the potentially protective role that GDF-15 might have in inflammatory regulation (10), it may provide a novel candidate biomarker for predicting functional decline across multiple organ systems.

We therefore tested whether GDF-15 was (a) associated with baseline measures of vascular and physical function and (b) predicted future decline in vascular and physical function, in a cohort of older patients with isolated systolic hypertension.

## Methods

We conducted analyses using samples and data collected prospectively as part of the vitamin D in isolated systolic hypertension (VitDISH) randomised controlled trial, which tested the effect of vitamin D supplementation on blood pressure in patients aged 70 and over with isolated systolic hypertension [10]. Participants were randomised into VitDISH if they had a baseline systolic BP of >140 mmHg but <180 mmHg, a baseline diastolic BP of <90 mmHg and a baseline 25OHD level of <75 nmol/L. Participants received 100,000 units of oral vitamin D3 or placebo every 3 months for a year in the trial, which showed no significant effect of vitamin D supplementation on any outcome measured. The trial was approved by the Fife and Forth Valley National Health Service Research Ethics Committee [10].

Outcome measurements from the VitDISH trial available for this analysis were BMI, office systolic and diastolic blood pressure, B-type natriuretic peptide, C-reactive protein, serum-adjusted calcium, glucose, cholesterol, parathyroid hormone and 25OHD levels, submaximal exercise capacity measured using the 6-min walk, hospital anxiety and depression scores (HADS), endothelial function measured using brachial artery flow-mediated dilatation (FMD), and carotid-radial pulse wave velocity using the Sphygmocor system as a measure of arterial stiffness. Baseline demographic data included age, gender and a past medical history of the following conditions: diabetes mellitus, myocardial infarction, angina, peripheral vascular disease and stroke/TIA.

Stored baseline serum samples were used for GDF-15 assays. Samples were allowed to clot at room temperature for 15 min, then spun at 3000 rpm for 10 min and stored in freezers (−20°C) for between three and a half to 5 years. GDF-15 was measured in duplicate using a Quantikine^®^ ELISA kit (R&D Systems, Inc., Minneapolis, MN, USA). The mean intra-assay coefficient of variation (CV) was 4.4 %, and the inter-assay CV was 19.8 %.

All statistical analyses were performed using IBM SPSS Statistics version 22.0 (IBM, New York, USA). Significance was defined as a two-sided ***p*** **<** 0.05. Baseline population characteristics are presented as frequencies and proportions, mean values with standard deviations or as medians with inter-quartile ranges (Table 1). As GDF-15, CRP and BNP levels were not normally distributed, results were log-transformed prior to analysis to enable parametric testing. Associations between baseline variables were examined using Pearson’s correlation coefficient for continuous variables, and with Student’s t-test for dichotomous variable. Multivariable linear regression models were used to determine associations between baseline log_10_GDF-15 levels and other baseline variables, and between baseline log_10_GDF-15 levels and changes in measures of vascular and physical health between baseline and 12 months. Both unadjusted and adjusted analyses were performed, with adjustment for baseline variables likely to influence either vascular health or physical function. The adjusted models were run both with and without age as a covariate, in case the strong correlation between age and log_10_GDF-15 masked a potentially important association.

**Table 1:** Baseline details and univariate associations with GDF-15 levels (*n* = 146) [11]

Factor	Baseline level	Correlation versus log_10_GDF-15	*p* for correlation
Mean age (years) (SD)	76.8 (4.7)	0.37*	<0.001
Body mass index (Kg/m^2^) (SD)	28.2 (4.8)	0.03	0.69
Median baseline GDF-15 (pg/mL) (IQR)	400 (246)	–	–
Systolic blood pressure (mmHg) (SD)	162 (11)	0.01	0.91
Diastolic blood pressure (mmHg) (SD)	78 (7)	−0.17*	0.04
Adjusted calcium (mmol/L) (SD)	2.31 (0.07)	−0.07	0.42
Glucose (mmol/L) (SD)	5.3 (0.8)	0.10	0.26
Total cholesterol (mmol/L) (SD)	4.9 (1.1)	−0.33*	<0.001
Parathyroid Hormone (pmol/L) (SD)	5.4 (2.5)	0.07	0.42
Median C-reactive protein (mg/L) (IQR)	1.7 (0.8 – 3.9)	0.04	0.63
Median BNP (pg/mL) (IQR)	31 (14 – 81)	0.11	0.18
25OHD (nmol/L) (SD)	44 (15)	−0.11	0.17
Pulse wave velocity (m/s) (SD)	8.8 (1.2)	−0.03	0.71
FMD of brachial artery (%) (SD)	5.2 (2.7)	0.06	0.51
Mean HADS score (anxiety) (SD)	4.2 (2.7)	−0.03	0.69
Mean HADS score (depression) (SD)	3.4 (2.4)	0.26*	0.001
Six-minute walking distance (m) (SD)	402 (99)	−0.37*	<0.001

		Mean log_10_GDF-15 Present versus absent	*p* for comparison
Male, N. (%)	77 (52)	2.63 versus 2.56*	0.01
Diabetes mellitus, N. (%)	17 (12)	2.74 versus 2.58*	<0.001
Myocardial infarction, N. (%)	9 (6)	2.74 versus 2.59*	0.02
Angina/PTCA/CABG, N. (%)	33 (22)	2.66 versus 2.58*	0.02
Peripheral vascular disease, N. (%)	10 (7)	2.63 versus 2.60	0.66
Stroke/TIA, N. (%)	16 (11)	2.64 versus 2.59	0.33

*BNP* b-type natriuretic peptide, *CABG* coronary artery bypass graft, *FMD* flow-mediated dilatation *GDF-15* growth differentiation factor-15, *HADS* hospital anxiety and depression score, *HDL* high-density lipoprotein, *IQR* interquartile range, *LDL* low-density lipoprotein, *PTCA* percutaneous transluminal coronary angioplasty, *SD* standard deviation, *TIA* transient ischaemic attack

* *p* < 0.05

## Results

 Sufficient stored baseline samples were available for 147/159 participants, who had been randomised to VitDISH. Analyses were conducted on 146 of thesσe, as one sample was excluded due to >30 % difference between duplicates on GDF-15 testing. Table 1 shows the baseline population characteristics for these individuals, along with baseline associations between each variable and log_10_GDF-15 levels. Baseline log_10_GDF-15 levels were significantly associated with increasing age, total cholesterol levels, diastolic BP and HADS depression scores. Independent sample t-tests showed significantly higher baseline log_10_GDF-15 in males, and those with a history of diabetes, myocardial infarction and angina. No significant associations were demonstrated with baseline measures of vascular function. Baseline multivariable linear regression showed GDF-15 levels remained associated only with age (beta = 0.29, *p* < 0.001).

Baseline GDF-15 correlated significantly with 12-month changes in LDL cholesterol (*r* = 0.06, *p* = 0.019) and 6-min walk distance (*r* = 0.23, *p* = 0.009), but not with other measures. Multivariable linear regression (Table 2) showed that higher baseline GDF-15 was associated with steeper decline in 6-min walk distance at 12 months after adjustment, but no relationship with changes in blood pressure, nor markers of vascular health, was evident.

**Table 2:** Association of baseline log_10_GDF-15 Levels with 12-month changes in vascular and physical health

Variable	Unadjusted		Adjusted model excluding age^a^		Adjusted model including age^a^	
	Beta	*p*	Beta	*p*	Beta	*p*
Systolic blood pressure	0.02	0.87	−0.11	0.30	−0.11	0.28
Diastolic blood pressure	0.02	0.80	−0.14	0.17	−0.16	0.14
Pulse wave velocity	−0.06	0.56	−0.04	0.65	−0.05	0.64
FMD of brachial artery	−0.03	0.78	0.04	0.55	0.05	0.54
B-type natriuretic peptide	0.10	0.43	0.06	0.75	0.05	0.78
Six-minute walk distance	−0.15	0.11	−0.26*	0.02	−0.27*	0.01

*FMD* flow-mediated dilatation, *GDF* growth differentiation factor

* *p* < 0.05

^a^Adjusted for baseline vascular disease, blood pressure, cholesterol, FMD, PWV, BNP, glucose, calcium, PTH, CRP, 25OHD, 6-min walk and sex

## Discussion

Our results suggest that in older patients with hypertension, GDF-15 may be an independent predictor of declining physical function, but does not predict change in vascular function. Whilst our investigation supported previous associations of GDF-15 levels with cardiovascular risk factors and the presence of vascular disease, the responsible mechanisms are not clear, as no correlation was seen between GDF-15 and endothelial function nor arterial stiffness (two key pathophysiological intermediaries for causing vascular disease).

The lack of correlation between GDF-15 and both endothelial function and arterial stiffness may be explained by numerous factors. Firstly, both FMD and PWV measure macrovascular, rather than microvascular, function. The PIVUS study also noted no correlation with brachial artery FMD, but observed significant correlations between GDF-15 and endothelium-dependent and endothelium-independent microvascular vasodilatation [11]. Another proposed pathway linking GDF-15 to CVD is the phosphoinositide 3-kinase (PI3k)/protein kinase Cζ (PKCζ)/specificity protein 1 (SP1) pathway [12]. This increases ABCA1 gene expression and causing greater macrophage cholesterol efflux, perhaps limiting foam cell formation to confer atherosclerosis protection. This is substantiated by the correlations between GDF-15 and cholesterol levels evidenced by both our results and those of previous studies [11, 13]. Finally, the macrovascular disease mechanisms in older people (for example, the relevance of vascular calcification) may not be associated with GDF-15 in the same way as changes seen in previously studied younger cohorts.

GDF-15 is an autocrine inhibitor of inflammation, released in response to pro-inflammatory cytokines such as IL-6 or TNF-α [14]. However, its mechanisms of action remain unclear due to the broad diversity of its functions [14]. Unlike traditional anti-inflammatory pathways, detailed molecular knowledge of GDF-15 signalling remains sparse and deserves further exploration. The previously well-established positive correlation between GDF-15 and age was corroborated by our results [13]. Numerous age-related conditions (atherosclerosis, chronic kidney disease, obesity and diabetes, cancer and cognitive impairment) are also associated with elevated GDF-15, making it a clinically relevant biomarker. Gene expression studies on hNAG-18 have shown that GDF-15 may influence age-associated pathology development through mechanisms related to obesity, appetite, insulin sensitivity, energy metabolism and mTOR activity [6]. These complex interactions represent pathways that may be implicated in the development of sarcopenia (reduced muscle strength and functional ability), which can impair physical function in old age. Excessively elevated GDF-15 levels, as seen in chronic inflammatory conditions, cancer and ageing, can cause cachexia. These effects seem to be mediated via neurones (in the hypothalamus and area postrema and nucleus tractus solitarius of the brainstem) that regulate appetite [15], and ERK1 and 2, neuropeptide Y and melanocortin signalling [16], confirming the role of protein-wasting and sarcopenia in contributing to worsening 6-min walking distances.

It is also important to acknowledge the potential effects of pre-existing risk factors and co-morbidities on physical function. Our results showed that those with higher baseline GDF-15 levels were more likely to be male, and have a past medical history of diabetes mellitus, myocardial infarction and/or ischaemic heart disease. Given the large degree of overlap between these demographics and GDF-15 levels, it is currently impossible to extricate the effects of each individual factor. Moreover, the lifestyle factors associated with these conditions (such as lack of exercise, poor diet and smoking), and the restrictions on exercise tolerance and activity levels that accompany conditions such as ischaemic heart disease, are also likely to limit physical function. Despite this assortment of contributory factors, it is possible that GDF-15 may serve as a unifying, objective marker for their cumulative effect on physical function.

### Strengths, limitations and future work

Our analysis is strengthened by the availability of detailed phenotypic data on a range of vascular, physical and psychological measures, and by the availability of 1-year follow-up data on most participants, enabling adjustment for several important confounders.

However, a number of limitations deserve comment. The study population was limited by its size and by all participants being of white ethnicity. We therefore cannot assume that they are representative of the wider elderly population. The age of our cohort and the presence of hypertension inevitably limit the scope for correlation analyses to detect links between measures of vascular function and GDF-15 levels, due to the limited range of these variables. A population-based study would likely increase the range of FMD, PWV and GDF-15 values, enhancing the ability to detect associations. Moreover, a wider panel of dependent variables (including measures of microvascular function and frailty—e.g. weight loss, grip strength, sit–stand time and ability to carry out activities of daily living) would be of considerable value in future studies. Studying other clinical outcomes such as time to hospital readmission or mortality data would also be of interest. However, meaningful analysis of such outcomes will require larger study sample sizes and longer follow-up than was available for this analysis.

Knowledge of the biological mechanisms of action of GDF-15 remains incomplete. Future studies should attempt not only to determine whether GDF-15 predicts adverse outcomes in a wider range of older people, but to ascertain whether GDF-15 is part of a causal pathway leading to sarcopenia and dysfunction of other organ systems, whether raised GDF-15 levels actively contribute to protective responses to pathology, or whether raised levels are a bystander marker of other pathological processes (e.g. oxidative stress or other inflammatory signals). Finally, the effect of interventions known to improve physical function (e.g. exercise training) on GDF-15 levels may help answer these questions.

## Conclusion

Baseline GDF-15 may be associated with walk distance and predicts decline in walk distance, but is not associated with vascular function in older patients with hypertension. These findings may enable the development of new ways to detect those at risk of functional decline, as well as suggesting new pathways to target the inflammatory causes and consequences of ageing.
